# The associations between some biological markers, obesity, and cardiovascular risk in Slovenian children and adolescents

**DOI:** 10.1186/s12887-020-1978-5

**Published:** 2020-02-21

**Authors:** Nataša Marčun Varda, Martina Medved, Laura Ojsteršek

**Affiliations:** 1 Department of Pediatrics, University Medical Center Maribor, Ljubljanska 5, 2000 Maribor, Slovenia; 2 University Medical Center Maribor, Ljubljanska ulica 5, 2000 Maribor, Slovenia

**Keywords:** Leptin, Ghrelin, Adiponectin, Body mass index, Hypertension, Hyperlipidemia

## Abstract

**Introduction:**

The occurrence of cardiovascular diseases and metabolic disorders steadily increases with the body mass index (BMI). Since the latter is not the best and earliest indicator of obesity and cardiovascular risk, the aim of the study was to evaluate some potential biological markers that would allow us to detect children and adolescents at higher risk at an early stage.

**Methods:**

A sample of 330 children and adolescents were included in the study and divided into four groups: obese patients with hypertension, normal-weight patients with hypertension, patients with mildly elevated lipids and a control group of healthy children and adolescents. Some clinical parameters (age, body weight, body height, BMI, waist circumference, hip circumference, blood pressure), biochemical parameters (glucose, total cholesterol, triglycerides, HDL, LDL, apolipoprotein A1, homocysteine) and biological markers of obesity (ghrelin, adiponectin, leptin) were evaluated.

**Results:**

Ghrelin and adiponectin were found to have a strong negative statistically significant correlation with BMI in all three observed groups (*p* < 0.001), but not in the control group (*p* = 0.053 and *p* = 0.316, respectively). In addition, leptin had a strong positive statistically significant correlation with BMI in all four groups (*p* < 0.001 for the research groups, *p* = 0.009 for the controls). In the group of obese patients with hypertension, statistically significant differences in all three markers of obesity were found in comparison to the control group (*p* < 0.001 for all markers). In the group of patients with mildly elevated lipids, ghrelin and leptin were significantly different (*p* = 0.002 and *p* < 0.001, respectively). In the group of normal-weight hypertensive patients, only values of ghrelin were different compared to the control group (*p* = 0.001).

**Conclusion:**

In the research groups, significant differences were found in clinical, biochemical and biological parameters compared to the control group. The observed biological markers of obesity are useful early markers for identifying groups of patients that are at cardiovascular risk.

## Background

Obesity is a common disease in childhood that represents an epidemiological problem in some countries, including Slovenia [1, 2]. It is one of the significant cardiovascular risk factors associated with higher cardiovascular morbidity and other risk factors like hypertension and hyperlipidemia [1]. Moreover, it has been shown that obese children are more likely to stay overweight in adulthood and to experience cardiovascular diseases at an early age compared to their peers with normal weight [1]. Both hypertension and hyperlipidemia can also occur in children and are associated with the occurrence and cardiovascular outcomes in adulthood [3]. Numerous studies have found that cardiovascular morbidity and mortality increase with body mass index (BMI [2, 4]. Although BMI is used as a measure of obesity, it is not sensitive enough to detect early fat accumulation and early cardiovascular risk [5–7]. However, one of the crucial aims of preventive pediatrics is to detect children with higher cardiovascular risk at an early stage. Hence research is directed towards finding early markers of cardiovascular risk, including potential biological markers like leptin, ghrelin and adiponectin [4].

There has been a considerable amount of research on leptin, and less on adiponectin and ghrelin [8–14]. Obese children have high concentrations of leptin and low values of adiponectin, but their codependency has not yet been proven [8, 9]. In some studies, their correlations with anthropometrical parameters have been shown [8, 11]. Recently, the associations with metabolic syndrome have also been found, suggesting their potential for cardiovascular risk prediction [12]. In a few studies published on ghrelin, lower values have been revealed in obese children [10, 14]. In addition, its association with some cardio-metabolic factors has been shown [15].

The aim of the present study was to evaluate the potential role of some biological obesity markers as early cardiovascular risk factors. In addition, their correlations with BMI, a known marker of obesity, were investigated in three study groups (the hypertensive group, the obese hypertensive group, and the mild hyperlipidemia group).

## Materials and methods

The study included 330 children and adolescents, aged from 8 months to 18 years, who were treated at our Department of Pediatrics. Patients were recruited prospectively, according to their risk factors. Body height and weight were measured to determine BMI. The Riva-Rocci method was used to measure blood pressure with appropriate cuff size (the bladder length covering 80%–100% of the circumference of the arm, the width at least 40%) and the first (phase I Korotkoff) and last (phase V Korotkoff) audible sounds to identify systolic and diastolic blood pressure, respectively. Fasting blood samples were used to assess leptin, adiponectin and ghrelin values. The levels of total adiponectin and leptin in the blood were measured using the quantitative sandwich enzyme immunoassay technique and the ghrelin level was measured using the sandwich ELISA method [16–18]. In addition, values of other biochemical cardiovascular risk factors were also measured from fasting blood serum using standard procedures (glucose − enzymatic hexokinase method, Siemens Dimension Vista; total cholesterol − cholesterol esterase enzymatic method, Siemens Dimension Vista; HDL and LDL cholesterol − homogenous direct method, Siemens Dimension Vista; triglycerides − enzymatic method, Siemens Dimension Vista; apolipoprotein A1 − nephelometric method, Siemens Healthineers Pro-Spec nephelometer; homocysteine − chemiluminiscent microparticle immunoassay, Abbott Architect 4000 SR) [19].

Children and adolescents were classified into four groups: obese patients with hypertension (92 patients), normal-weight patients with hypertension (104 patients), patients with mildly elevated lipids (99 patients) and a control group of 35 healthy children and adolescents. Obesity was defined as a BMI above the 95th percentile.

The data were statistically analyzed using IBM SPSS Statistics 22.0 with descriptive statistics (the mean values with the standard deviations), the Analysis of variance (ANOVA) tests with post hoc Bonferroni adjustment and bivariate correlations. The strength of correlation was defined by the Pearson coefficient (r), where a weak correlation ranged between 0 and 0.3, a moderate one with r between 0.3 and 0.7, and a strong correlation with r between 0.7 and 1. A significant statistical difference was defined as *p* < 0.05.

The research was approved by the institutional Ethical Committee.

## Results

The characteristics of the four examined groups are shown in Table 1. Statistically significant differences according to the ANOVA post hoc Bonferroni adjusted tests between the control and the research groups are highlighted. Of note are the elevated values of both systolic and diastolic blood pressure that were also found in children with hyperlipidemia. There was no statistical difference in diastolic blood pressure in the other two research groups. In addition to children with elevated lipids, differences in some lipids were also found in the group of obese hypertensive children.

**Table 1:** Comparison of clinical and biochemical parameters between the control group and the group of obese patients with hypertension, the group of patients with mildly elevated lipids and the group of patients with normal-weight hypertension

Parameters/Mean ± SD (*p*-value)	Control group (*N* = 35)	Obese patients with hypertension (*N* = 92)	Patients with mildly elevated lipids (*N* = 99)	Normal-weight patients with hypertension (*N* = 104)
Age (years)	11.3 ± 4.6	12.4 ± 4.3 (N)	11.3 ± 3.8 (N)	13.3 ± 4.6 (N)
Body weight (kg)	45.9 ± 20.2	75.5 ± 26.3 (< 0.001)	73.4 ± 31.8 (< 0.001)	58.4 ± 22.4 (N)
Body height (cm)	149.3 ± 24.4	159.9 ± 20.7 (N)	155.4 ± 22.0 (N)	161.3 ± 25.7 (N)
BMI (kg/m^2^)	19.5 ± 3.9	28.0 ± 5.1 (< 0.001)	28.5 ± 7.9 (< 0.001)	20.8 ± 3.8 (0.004)
Waist circumference (cm)	69.5 ± 11.9	92.6 ± 15.6 (< 0.001)	93.4 ± 21.6 (< 0.001)	75.2 ± 12.8 (0.03)
Hip circumference (cm)	82.3 ± 12.4	101 ± 15.6 (< 0.001)	99.7 ± 20.0 (< 0.001)	88.3 ± 15.0 (N)
Systolic blood pressure (mmHg)	115.5 ± 9.5	128.3 ± 9.5 (< 0.001)	123.0 ± 15.5 (0.009)	126.8 ± 11.5 (< 0.001)
Diastolic blood pressure (mmHg)	68.5 ± 6.8	71.4 ± 8.1 (N)	75.8 ± 11.8 (< 0.001)	70.6 ± 7.9 (N)
Glucose (mmol/l)	4.9 ± 0.9	4.9 ± 0.7 (N)	4.8 ± 0.5 (N)	4.7 ± 0.4 (N)
Total cholesterol (mmol/l)	4.1 ± 0.7	4.4 ± 0.5 (N)	4.6 ± 1.0 (0.045)	4.2 ± 0.93 (N)
Triglycerides (mmol/l)	0.96 ± 0.5	1.3 ± 0.96 (0.059)	1.18 ± 0.6 (N)	0.91 ± 0.4 (N)
HDL cholesterol (mmol/l)	1.6 ± 0.5	1.2 ± 0.3 (< 0.001)	1.3 ± 0.4 (0.002)	1.5 ± 0.4 (N)
LDL cholesterol (mmol/l)	2.3 ± 0.6	2.8 ± 0.6 (0.030)	2.9 ± 0.9 (0.005)	2.7 ± 1.2 (N)
Apolipoprotein A1 (g/l)	1.5 ± 0.3	1.4 ± 0.2 (N)	1.4 ± 0.2 (N)	1.5 ± 0.24 (N)
Homocysteine (μmol/l)	8.6 ± 2.4	10.4 ± 5.1 (N)	7.9 ± 2.2 (N)	10.1 ± 4.0 (N)

*BMI* body mass index, *N* nonsignificant

The correlations with BMI are displayed in Table 2, confirming correlations for all three research groups.

**Table 2:** Correlations of ghrelin, adiponectin and leptin with body mass index in all observed groups (r and *p* values are presented)

Parameter/correlation coefficient r (*p*-value)	Control group of children and adolescents	Obese patients with hypertension	Patients with mildly elevated lipids	Normal-weight patients with hypertension
Ghrelin (pg/ml)	−0.356 (0.058)	− 0.433 (< 0.001)	−0.577 (< 0.001)	− 0.4756 (< 0.001)
Adiponectin (ng/ml)	−0.179 (0.353)	− 0.467 (< 0.001)	−0.568 (< 0.001)	− 0.508 (< 0.001)
Leptin (pg/ml)	0.478 (0.009)	0.463 (< 0.001)	0.685 (< 0.001)	0.371 (< 0.001)

**Fig. 1:**
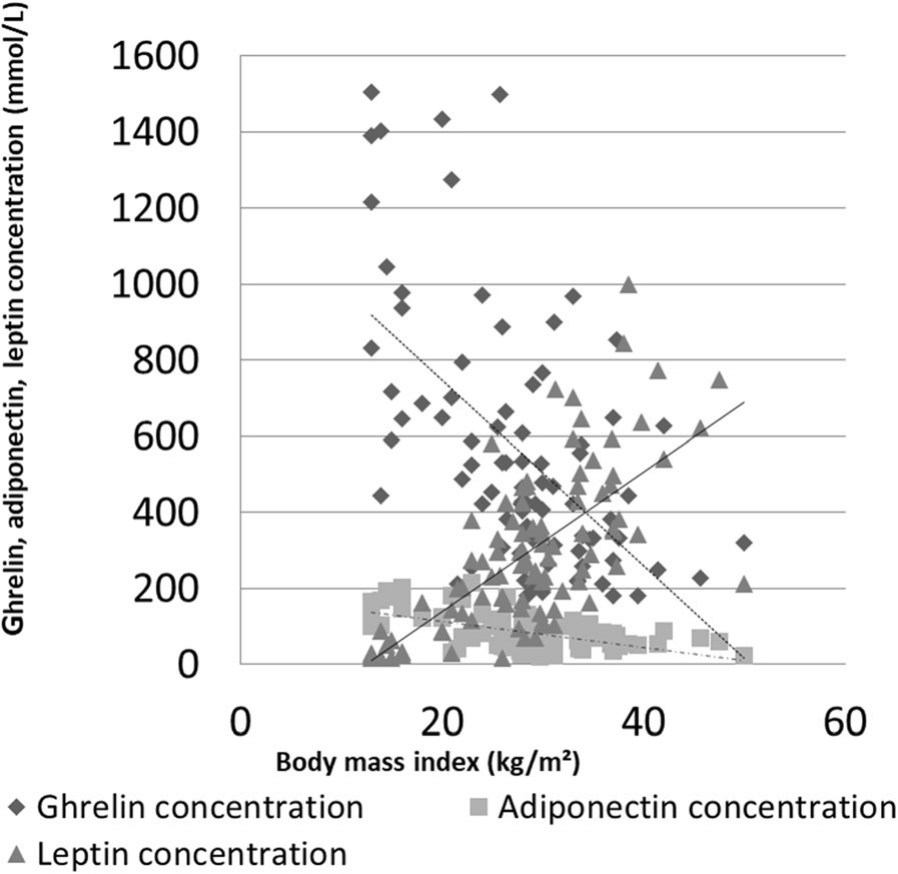
Correlations between ghrelin, adiponectin and leptin and body mass index in the group of obese patients with hypertension

Correlations between BMI and all biomarkers in obese hypertensive patients are presented in Fig. 1.

In Table 3, the mean values of individual biological markers are highlighted and compared to the control group. Differences were found in all the study groups.

**Table 3:** Comparison of biological markers of obesity (ghrelin, adiponectin and leptin) between the control group of children and adolescents, the group of obese patients with hypertension, the group of patients with mildly elevated lipids and the group of patients with normal-weight hypertension

Parameters/Mean ± SD (*p*-value)	Control group of children and adolescents (*N* = 35)	Obese patients with hypertension (*N* = 92)	Patients with mildly elevated lipids (*N* = 99)	Normal-weight patients with hypertension (*N* = 104)
Ghrelin (pg/mL)	865.5 ± 794.0	432.7 ± 216.9 (< 0.001)	557.1 ± 334.5 (< 0.001)	559.0 ± 371.6 (0.001)
Adiponectin (ng/ml)	103.7 ± 50.9	63.8 ± 38 (< 0.001)	87.1 ± 47.4 (N)	83.8 ± 40.4 (N)
Leptin (pg/ml)	95.4 ± 111.1	278.2 ± 200.4 (< 0.001)	295.7 ± 211.3 (< 0.001)	76.1 ± 94.5 (N)

**Table 4:** Correlations of ghrelin, adiponectin and leptin with other investigated parameters in the groups of obese hypertensive patients, patients with mildly elevated lipids and normal-weight patients with hypertension (r and p values are presented)

	Obese patients with hypertension			Patients with mildly elevated lipids			Normal-weight patients with hypertension		
Parameter/Correlation coefficient r (*p*-value)	Ghrelin	Adiponectin	Leptin	Ghrelin	Adiponectin	Leptin	Ghrelin	Adiponectin	Leptin
Age (years)	-0.452 (*p*<0.001)	-0.519 (*p*<0.001)	0.132 (*p*=0.212)	-0.633 (*p*<0.001)	-0.562 (*p*<0.001)	0.407 (*p*<0.001)	-0.585 (*p*<0.001)	-0.492 (*p*<0.001)	0.059 (*p*=0.555)
Body weight (kg)	-0.486 (*p*<0.001)	-0.516 (*p*<0.001)	0.219 (*p*=0.037)	-0.638 (*p*<0.001)	-0.583 (*p*<0.001)	0.567 (*p*<0.001)	-0.577 (*p*<0.001)	-0.556 (*p*<0.001)	0.048 (*p*=0.630)
Body height (cm)	-0.496 (*p*<0.001)	-0.535 (*p*<0.001)	0.059 (*p*=0.578)	-0.643 (*p*<0.001)	-0.575 (*p*<0.001)	0.375 (*p*<0.001)	-0.622 (*p*<0.001)	-0.492 (*p*<0.001)	-0.097 (*p*=0.329)
BMI (kg/m²)	-0.433 (*p*<0.001)	-0.467 (*p*<0.001)	0.463 (*p*<0.001)	-0.577 (*p*<0.001)	-0.568 (*p*<0.001)	0.685 (*p*<0.001)	-0.475 (*p*<0.001)	-0.508 (*p*<0.001)	0.371 (*p*<0.001)
Waist circumference (cm)	-0.453 (*p*<0.001)	-0.498 (*p*<0.001)	0.287 (*p*=0.007)	-0.634 (*p*<0.001)	-0.576 (*p*<0.001)	0.610 (*p*<0.001)	-0.587 (*p*<0.001)	-0.561 (*p*<0.001)	0.172 (*p*=0.092)
Hip circumference (cm)	-0.526 (*p*<0.001)	-0.519 (*p*<0.001)	0.350 (*p*=0.001)	-0.668 (*p*<0.001)	-0.582 (*p*<0.001)	0.618 (*p*<0.001)	-0.637 (*p*<0.001)	-0.489 (*p*<0.001)	0.149 (*p*=0.174)
Systolic blood pressure (mmHg)	-0.407 (*p*<0.001)	-0.312 (*p*=0.003)	-0.202 (*p*=0.055)	-0.387 (*p*<0.001)	-0.319 (*p*=0.002)	0.182 (*p*=0.078)	-0.264 (*p*=0.007)	-0.188 (*p*=0.049)	-0.056 (*p*=0.571)
Diastolic blood pressure (mmHg)	-0.139 (*p*=0.188)	0.084 (*p*=0.429)	-0.086 (*p*=0.419)	-0.353 (*p*=0.001)	-0.345 (*p*=0.001)	0.336 (*p*=0.001)	0.041 (*p*=0.680)	0.184 (*p*=0.061)	0.085 (*p*=0.390)
Ghrelin (pg/ml)	/	0.376 (*p*<0.001)	-0.035 (*p*=0.744)	/	0.483 (*p*<0.001)	-0.391 (*p*<0.001)	/	0.420 (*p*<0.001)	-0.141 (*p*=0.154)
Adiponectin (ng/ml)	0.376 (*p*<0.001)	/	-0.105 (*p*=0.326)	0.483 (*p*<0.001)	/	-0.266 (*p*=0.011)	0.420 (*p*<0.001)	/	0.000 (*p*=0.999)
Leptin (pg/ml)	-0.035 (*p*=0.744)	-0.105 (*p*=0.326)	/	-0.391 (*p*<0.001)	-0.266 (*p*=0.011)	/	-0.141 (*p*=0.154)	0.000 (*p*=0.999)	/
Glucose (mmol/l)	-0.150 (*p*=0.165)	-0.072 (*p*=0.510)	0.114 (*p*=0.292)	0.010 (*p*=0.926)	-0.066 (*p*=0.544)	-0.032 (*p*=0.759)	-0.298 (*p*=0.002)	-0.021 (*p*=0.832)	0.178 (*p*=0.074)
Total cholesterol (mmol/l)	-0.123 (*p*=0.247)	0.073 (*p*=0.496)	-0.068 (*p*=0.522)	0.174 (*p*=0.114)	0.409 (*p*<0.001)	-0.045 (*p*=0.663)	0.097 (*p*=0.330)	0.204 (*p*=0.039)	0.273 (*p*=0.005)
Triglycerides (mmol/l)	-0.185 (*p*=0.08)	-0.144 (*p*=0.180)	0.015 (*p*=0.890)	-0.097 (*p*=0.379)	-0.240 (*p*=0.022)	0.075 (*p*=0.463)	-0.257 (*p*=0.009)	-0.174 (*p*=0.078)	0.411 (*p*<0.001)
HDL cholesterol (mmol/l)	0.311 (*p*=0.003)	0.422 (*p*<0.001)	0.00 (*p*=0.997)	0.216 (*p*=0.049)	0.413 (*p*<0.001)	-0.173 (*p*=0.089)	0.216 (*p*=0.028)	0.421 (*p*<0.001)	0.066 (*p*=0.504)
LDL cholesterol (mmol/l)	-0.174 (*p*=0.102)	-0.086 (*p*=0.424)	-0.031 (*p*=0.768)	0.065 (*p*=0.561)	0.332 (*p*=0.001)	0.009 (*p*=0.929)	0.026 (*p*=0.792)	0.068 (*p*=0.498)	0.170 (*p*=0.087)
Apolipoprotein A1 (g/l)	0.182 (*p*=0.1)	0.366 (*p*=0.001)	-0.051 (*p*=0.648)	0.145 (*p*=0.201)	0.351 (*p*=0.001)	-0.083 (*p*=0.435)	0.203 (*p*=0.048)	0.387 (*p*<0.001)	0.185 (*p*=0.072)
Homocysteine (μmol/l)	-0.276 (*p*=0.011)	-0.277 (*p*=0.012)	-0.098 (*p*=0.383)	-0.238 (*p*=0.222)	-0.310 (*p*=0.108)	-0.079 (*p*=0.690)	-0.360 (*p*<0.001)	-0.393 (*p*<0.001)	0.183 (*p*=0.080)

Correlation tests were made between researched biological markers (ghrelin, adiponectin, leptin) and other parameters in all the study groups (Table 4). Ghrelin and adiponectin significantly negatively correlated with age, anthropometric measurements including BMI, and systolic blood pressure in all three research groups. Diastolic blood pressure correlated negatively with both markers only in the group with mildly elevated lipids. Additionally, significant negative correlations were found between both markers and leptin in the group with mildly elevated lipids, between ghrelin and glucose, triglycerides and apolipoprotein A1 in the group with normal-weight hypertension, between both markers and homocysteine in both obesity-related hypertension and normal-weight hypertension. Ghrelin and adiponectin correlated positively with HDL-cholesterol in all three research groups. Furthermore, adiponectin correlated negatively with triglycerides and positively with both LDL-cholesterol and total cholesterol in the group with mildly elevated lipids. The positive correlation between adiponectin and both apolipoprotein A1 and ghrelin was also shown in all three groups.

The associations between leptin and researched parameters were less often significant. The positive correlations between leptin and all anthropometric parameters were found in both the obese hypertensive group and the mild hyperlipidemia group. In addition, leptin correlated positively with BMI, total cholesterol and triglycerides in the group with normal-weight hypertension, and with age, diastolic blood pressure, ghrelin and adiponectin in the mild hyperlipidemia group.

To emphasize, BMI was the only parameter that correlated significantly with all three biological markers, ghrelin, adiponectin and leptin, in all three research groups. Of note is the negative correlation found between systolic blood pressure and both ghrelin and adiponectin in all three research groups. For diastolic blood pressure, a correlation with all obesity markers was found only in the hyperlipidemia group. In addition, a positive correlation between ghrelin and adiponectin was shown in all the research groups.

**Fig. 2:**
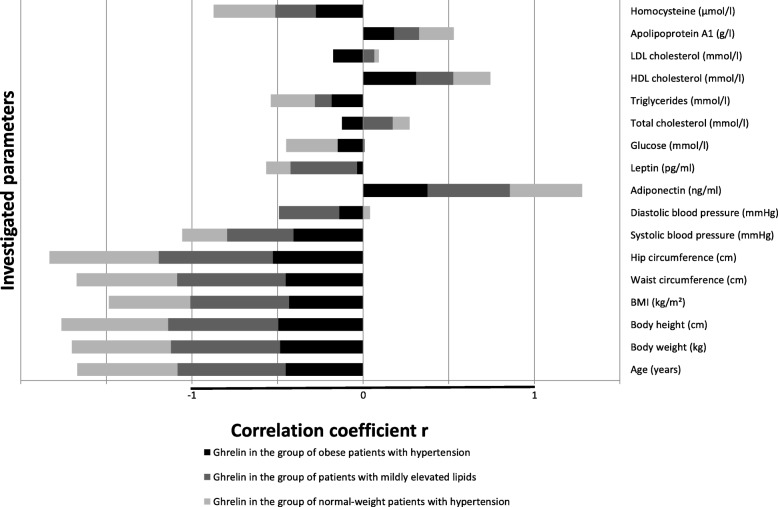
Correlations of ghrelin with other investigated parameters in the groups of obese hypertensive patients, patients with mildly elevated lipids and normal-weight patients with hypertension

**Fig. 3:**
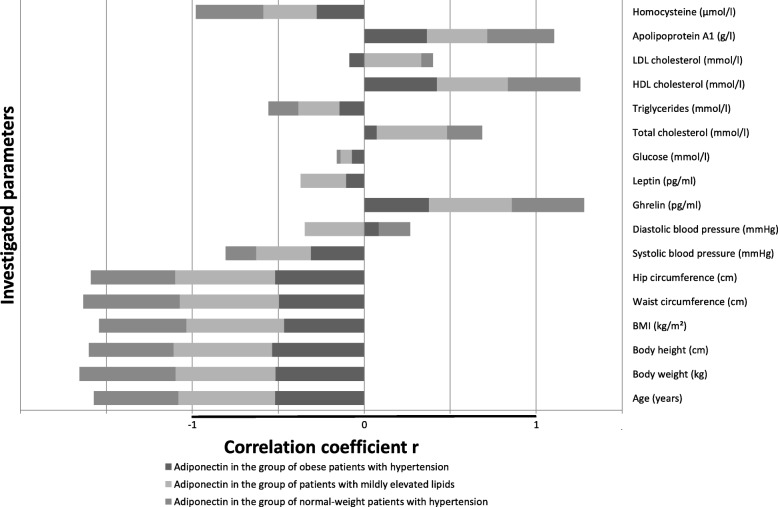
Correlations of adiponectin with other investigated parameters in the groups of obese hypertensive patients, patients with mildly elevated lipids and normal-weight patients with hypertension

**Fig. 4:**
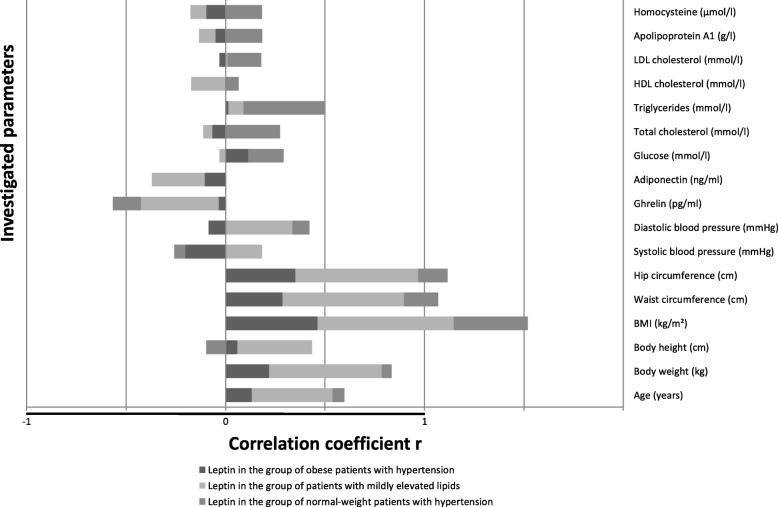
Correlations of leptin with other investigated parameters in the groups of obese hypertensive patients, patients with mildly elevated lipids and normal-weight patients with hypertension

In Figs. 2, 3 and 4, correlation factors between the biomarkers and other parameters are presented for all three research groups.

## Discussion

In the current study, the potential role of some biological obesity markers as early cardiovascular risk factors was investigated in three groups of children and adolescents, patients with obesity-related hypertension, patients with mildly elevated lipids and patients with normal-weight hypertension. In all the research groups, their correlations with BMI, a known marker of obesity, were confirmed. The most promising biological marker was found to be ghrelin, proposed for early obesity and cardiovascular risk detection.

Comparison of individual groups with the control group of healthy children and adolescents showed no significant age differences, although there was a wide age range. In addition to significant differences in anthropometrical parameters in the obese hypertensive group, the results also showed differences in BMI as well as waist circumference in the other two research groups, indicating an impact of environmental factors [20]. Data from the group of patients with hypertension showed that they had mild hypertension, as the mean values of systolic blood pressure were 126.8 mmHg in normal-weight patients with hypertension and 128.3 mmHg in obese patients with hypertension. Similarly, the results of the lipid profile in the hyperlipidemia group showed that patients with mild metabolic disturbance were included. These are precisely the groups that require early markers of cardiovascular risk, indicating the importance of our results. Except for the group of patients with hypercholesterolemia, no statistically significant differences in diastolic blood pressure were found, confirming the findings in other studies investigating children with mild cardiovascular disturbances [21, 22].

Among biochemical parameters, the average values of total cholesterol, LDL cholesterol and triglycerides were higher in the groups of obese patients with hypertension and those with elevated lipids. HDL cholesterol was lowest in obese patients with hypertension. These parameters were therefore confirmed as significant risk factors in the obesity-related hypertension group, which is in agreement with previous studies [20, 23]. Glucose and homocysteine concentrations were comparable in all groups and did not differ from the control group.

All three investigated groups showed a statistically significant difference in the ghrelin concentration compared to the control group. In contrast, a statistically significant difference in the adiponectin concentration was found only in the group of obese patients with hypertension. The ghrelin concentration was lowest in obese patients with hypertension, which shows its negative correlation with obesity and obesity-related hypertension. This is in agreement with the results of previously published studies, proving a compensatory decrease in ghrelin when adipose tissue increases [14, 24]. However, some previous studies in children found no correlation between ghrelin and obesity-related hypertension, a correlation that was recently shown in adults [15, 25]. In addition, our finding of the role of ghrelin in normal weight hypertension has not been found in any previous studies in children. The concentration of adiponectin was highest in the healthy population and lowest in obese patients with hypertension, which has also been found in other studies [12, 26]. Leptin has been shown to be significantly different in the groups of obese patients with hypertension and patients with mildly elevated lipids. Therefore, we confirmed previous research proving its role in obesity [26, 27].

All the analyzed groups had a statistically significant positive correlation between leptin and BMI and a negative correlation between ghrelin and adiponectin and BMI, which are in accordance with earlier studies, thus confirming their potential for the determination of the obesity risk [8, 15, 26].

The codependent work of leptin and adiponectin has not yet been proven. Some studies found that the leptin/adiponectin ratio could be an important parameter in obesity [8]. However, other studies have shown that the parameters of children’s body structure are related to leptin, but not to adiponectin [13]. In the present study, their correlation was found only in the group of patients with hyperlipidemia. The same was found for the correlation of leptin and ghrelin. Some studies proposed that leptin affects blood ghrelin levels since it has an impact on satiation, thus inhibiting ghrelin secretion [10]. It has also been proven that fasting plasma ghrelin values in obese patients inversely correlate with plasma leptin concentrations [28, 29]. However, recent research has proposed that the two systems might work independently of each other, with different pathophysiological mechanisms [30]. Our finding of a correlation between adiponectin and ghrelin in all three research groups will require confirmation in other studies and further research.

Subgroup analysis of included patients revealed a moderate negative statistical correlation between ghrelin/adiponectin and a positive correlation between leptin and anthropometrical parameters in the group of obese children and adolescents with hypertension. These results show their good potential for assessing obesity, which was also confirmed by other studies [24, 31]. In the present study, their observed correlation with systolic blood pressure indicates their role in hypertension risk. Recently, leptin has been found to play a role in obesity-related hypertension [32, 33] as has adiponectin in adult men [34].

In the group of children and adolescents with elevated lipids, a moderate negative statistical correlation was found between ghrelin and adiponectin and anthropometrical parameters as well as systolic and diastolic blood pressure, indicating their good predictive value in this group of patients. Leptin was found to have a moderate positive correlation with anthropometrical parameters as well as with diastolic blood pressure. Some previously published studies have found a correlation between lipid fractions and biological markers of obesity [27, 35]. To our knowledge, the correlation with blood pressure in such children has not been evaluated.

In the group of children and adolescents with normal-weight hypertension, the correlation between ghrelin, leptin and adiponectin and anthropometrical parameters has also been found. In a recent study, leptin was shown to correlate with blood pressure, but this was not confirmed in our study [32]. However, ghrelin and adiponectin have been confirmed as cardiovascular markers in normal-weight hypertensive patients, which has not been proven in children until now, although this correlation has been found in adult patients [36]. Interestingly, the finding of correlations between both ghrelin and adiponectin and homocysteine in patients with hypertension, obese and normal-weight, needs further investigations. In addition, their potential mechanisms of action, including inflammation [37] and other biological effects [38], have to be determined.

## Conclusion

Our study showed that biological markers of obesity have the potential to be used as early cardiovascular risk factors. It seems that the parameters are especially helpful in the obese population with hypertension. Ghrelin has been found to be a valid marker for all investigated groups and we recommend its use for the determination of cardiovascular risk. We have also confirmed that biological markers of obesity correlate with BMI, ghrelin and adiponectin negatively and leptin positively.

What this paper adds:

1. Biological markers of obesity are useful not only as markers of obesity but also as early cardiovascular risk markers.
2. Along with the large impact of leptin, ghrelin and adiponectin also play significant roles, with potential codependency.
3. The most promising biological marker in all three groups of children was found to be ghrelin, which we propose for the determination of cardiovascular risk.

## Data Availability

All data generated or analyzed during this study are included in this published article, except the correlations of the biological markers with other investigated parameters, presented as the Table 4.

## Abbreviations

ANOVA: Analysis of variance
BMI: Body mass index
N: Nonsignificant
SD: Standard deviation
